# Psychological Adjustment to Spousal Bereavement in Older Adults: A Systematic Review

**DOI:** 10.1177/00302228211043702

**Published:** 2021-09-01

**Authors:** Jack Purrington

**Affiliations:** Clinical Psychology Unit, The University of Sheffield, Department of Psychology, The University of Sheffield, Sheffield, United Kingdom

**Keywords:** Older adult, bereavement, spousal loss, psychological adjustment, adaptation

## Abstract

Research articles examining psychological adjustment to spousal bereavement in older adults (65+) were identified through searches on five electronical databases alongside forward citation and reference list searches. A total of 15 articles involving 686 unique participants were identified. Five characteristics were discovered which can facilitate and inhibit psychological adjustment to spousal bereavement in older adults: the pre-loss spousal relationship, social support, finding meaning and spirituality in loss, the surviving spouse’s personality traits, and death characteristics. These findings support that concepts of ‘meaning making’ and social support should be incorporated into therapeutic work with bereaved spouses to help facilitate psychological adjustment.

Identifying and understanding the factors associated with psychological adjustment to spousal bereavement in older adults is of intrinsic interest to all psychological practitioners (Carr, 2004; Galatzer-Levy & Bonanno, 2012; Laidlaw, 2015; Nesse et al., 2003; Parkes & Prigerson, 2013). Academic literature has been examining the concept of spousal bereavement for some time. Researchers such as Averill (1968) and Holmes and Rahe (1967) defined the processes of grief and widowhood as experiences and events which are amongst the most distressing a human being can encounter. Despite the extensive nature of interest within this research area, the increase in percentage of older adults worldwide (United Nation Department of Economic and Social Affairs Population Dynamics, 2019) justifies that continuous investigation and consolidation of information is required.

This notion has been reinforced within the context of the COVID-19 pandemic with most COVID-19 deaths worldwide being reported among individuals over the age of 65. It is not yet known how such an unprecedented rise in spousal bereavement will impact the psychological wellbeing of the cohort of surviving spouses nor how psychological and mental health support services will cope with the increased demand for access to service provision. However, information on psychological adjustment to bereavement in older adults could help mental health support services prepare for the high number of bereaved older adults during the pandemic. It is therefore critical to review what is known, what is not known, and what future research should consider in relation to the psychological adjustment of older adults following spousal bereavement.

An older adult can be defined as an individual aged 65 or older (Baltes & Smith, 2003; Laidlaw, 2015). Spousal bereavement can be defined as the enduring and holistic psychological and physical experiences of grief and adaptation following the death of a romantic partner (Bennett & Soulsby, 2012; Michael & Cooper, 2013; Stroebe et al., 2001). For many, spousal bereavement is an intense and distressing experience (Laidlaw, 2015; Michael & Cooper, 2013; Parkes & Prigerson, 2013; Stroebe et al., 2001). However, it can also promote personal growth as shown in Tedeschi et al.’s (1998) and Linley and Joseph’s (2004) work on growth following adversity. Successful psychological adjustment following spousal bereavement can be defined as an improvement in psychological functioning and a progress in adaptation compared to pre-loss levels (Calhoun & Tedeschi, 1998; Christopher, 2004; Linley and Joseph, 2004) alongside reduced levels of anxiety, depression, and grief-related distress (despair, intrusive thoughts, anger, shock, and yearning) (Carr, 2004; Ha, 2010; Kim, 2009; Nesse et al., 2003).

Two leading models theorising the concept of psychological adjustment after suffering are the shattered assumptions theory, see Janoff-Bulman (1992) and the organismic valuing theory, see Joseph and Linley (2005). Additionally, contemporary models of bereavement provide conceptualisations of loss-responses which align with principles of psychological adjustment.

The Dual Process Model (DPM; Stroebe & Schut, 2010) explains that after bereavement individuals oscillate between loss-oriented and restoration-oriented experiences of everyday life. Stroebe and Schut (2010) propose that in loss orientation bereaved individuals contend with grief reactions to loss, attempt to reorganise their attachment bond with the deceased, and resist the demands of restoration-orientated processes. Restoration-oriented experiences include attending to life changes, distraction from pain, and the negotiation of new roles, identities, and relationships to be congruent with life post-loss. A critical feature of the DPM is the oscillation between loss orientation and restoration orientation in which bereaved individuals modulate their experiences of distress by either attuning to the loss or to the demands of living. Neimeyer and Harris (2016) conceptualise this as the mourner ‘dosing’ their exposure to grief and address avoidance as having an important role in restoration. Therefore, bereaved individuals who can successfully regulate their post-loss experience by ‘dosing’ their exposure to grief are likely to adjust to bereavement more adaptively.

The Two-Track Model (TTM; Rubin, 1999) is a further model of bereavement which addresses two separate modes of coping: biopsychosocial and relational. The TTM posits that bereaved individuals face two ‘tracks’ concurrently. Mourners must attend to biopsychosocial post-loss experiences such as changes to sleep, appetite, and concentration, increases in anxiety, depression, and low self-esteem, and strained connections to others (Rubin et al., 2011). Alongside engaging in the relational adjustment to the deceased in which individuals recalibrate their relationship with their loved one via memories, stories, and rituals of remembrance. The TTM illustrates that adjustment to bereavement requires both the ability to cope with the biopsychosocial demands of bereavement and the appropriate navigation of the changing relationship with the deceased (Rubin et al., 2011).

Separate to these models Neimeyer (2001, 2002, 2014) has developed the theory of meaning reconstruction following bereavement which aligns closely with Janoff-Bulman’s (1992) shattered assumptions theory following traumatic experiences. The meaning reconstruction theory explains that experiences of bereavement are often incongruent with the survivor’s self-stories and life assumptions. Therefore, an important process of grief is to rebuild a coherent narrative which can accommodate the loss (Neimeyer, 2002). Neimeyer and Thompson (2014) suggested that mourners need to ‘make sense’ of the event story of loss alongside making sense of the relationship change to accommodate the loss into their life story and adjust to the bereavement.

However, despite the development of these theories of bereavement and the emergence of literature examining psychological adjustment to spousal loss, there has yet to be a review examining these two topics in older adults. Therefore, this systematic review aims to examine the available evidence on psychological adjustment to spousal bereavement in older adults. It is hoped that this review will help facilitate psychological and mental health support services to better recognise when positive psychological adjustment is likely to occur, to identify the potential barriers, mediators, and moderators of psychological adjustment, and to assist the process of adjustment.

## Method

The systematic review was completed in accordance with the preferred reporting items for systematic reviews guidelines (PRISMA; Moher et al., 2009).

### Search Strategy

Studies were collated across five electronic information databases: Web of Science, PsychInfo, Medline, Scopus and CINAHL. The search strategy followed similar systematic review protocols to Michael and Cooper (2013), Stahl and Schulz (2014), and Wang et al. (2018). Three groups of search terms were searched for within article titles, abstracts, and keywords, with the keywords and Boolean terms relating to (a) the population (Elderly OR older OR elder OR geriatric OR “late life” OR “older adults” OR ageing OR “elderly adults”); (b) the context of loss (Bereave* OR widow* OR grief OR “spousal loss” OR “spousal bereavement”); and (c) psychological adjustment (“Adaptive adjustment” OR “living well” OR growth OR “post-traumatic growth” OR “stress related growth” OR “benefit finding” OR “adversarial growth” OR “positive life change” OR “psychological adjustment”). The search commenced in January 2020 and terminated by 29th February 2020. For included studies, citation searches were undertaken utilising Google Scholar which revealed one additional study and reference lists were examined which identified another further study.

### Inclusion Criteria

The lead researcher screened studies against predetermined inclusion and exclusion criteria. Studies were included if they:

- were published between 1996 and 2020, as Tedeschi and Calhoun (1995) began publishing academic literature examining the concept of posttraumatic growth in 1995 it was deemed important to include research following this year.
- had samples collected from western cultures in the Northern Hemisphere to promote the generalisability of results.
- utilised quantitative measurements for both the dependent variable (psychological adjustment following spousal bereavement) and independent variables (characteristics of the bereaved and their behaviours) to facilitate an objective examination into the factors associated with psychological adjustment.
- clearly stated that at least one member of the spousal relationship was aged 65 years or above to ensure the older adult population was examined in line with Laidlaw’s (2015) ‘young old’ aging framework and Baltes and Smith’s (2003) ‘third age’ aging framework, and to conform to standard inclusion criteria in literature.
- were full-text articles or reviews with English versions available to comply with the lead researcher’s native language.

### Exclusion Criteria

Studies were excluded if they:

- gathered participants from collectivist cultures or locations in the Southern Hemisphere, as grief and bereavement traditions differ greatly across cultures (Rosenblatt, 2008).
- gathered participants from clinical samples or nursing homes, as pre-existing difficulties can significantly impact the recollection of events such as spousal bereavement (Carr, 2004; Teasdale et al., 1980).
- reported data exclusively on widows as this would have yielded an imbalanced dataset and produced a limited review insensitive to discussions relating to difference and diversity associated with gender.
- did not report psychological responses to spousal bereavement.

### Data Extraction

Data was extracted by the lead researcher utilising a structured table to guide this process. The focus of each study, design and sample, measures used, key findings, confounding variables, and overlap between studies were extracted with this information being presented in table 2.

**Table 2.** Description of Studies.

Study	Focus	Design and sample	Measures	Key findings	Confounds controlled for	Overlap between studies
Carr (2003)	Context of spousal bereavement.	Longitudinal cohort study. CLOC study: 151 Women 59 Men Age: M = 70	- CES-D adapted 9-items. - SCL90R adapted 10-items. - Grief items: yearning, anger, intrusive thoughts, & anxiety, taken from BI, PFL, & TRIG. - Quality of Death: acceptance (3-item), burden on surviving spouse (3-item), end of life care (2-item), social support prior to death (1-item), belief spouse lived a full life (1-item).	- Perception of spousal pain pre-death predicts yearning (*b* = .353;*p* < .05), intrusive thoughts (*b* = .448;*p* < 05), and anxiety (*b* = .301;*p* < 0.5). - Perception of physician negligence predicts anger (*b* = .698;*p* < .01).	- Depression. - Anxiety, - Religiosity, - Spousal health.	- Same sample as Carr (2004), different focus & measures. - Same sample as Pai and Carr (2010), different focus & measures.
Carr (2004)	Emotional and instrumental dependency.	Longitudinal cohort study. CLOC Study: Sample One: 210 bereaved 87 married controls Sample Two: 151 women 59 men Age: M = 70	- Self-Esteem (5-items). - Perceived Personal Growth (4-items). - CES-D adapted 9-items. - SCL90R adapted 10-items. - Instrumental Dependence (4-items) and Emotional Dependence (3-items) derived from DAS. - Friendship Support (2-items).	- Males instrumentally dependent on spouses pre-loss experience personal growth post-loss (*b* = -.38;*p* < .05). - Females emotionally dependent on spouses pre-loss experience personal growth post-loss (*b* = .05;*p* < 0.5).	- Pre-loss wellbeing. - Socio-economic status. - Physical health.	- Same sample as Carr (2003), different focus & measures. - Same sample as Pai and Carr (2010), different focus & measures.
Carr & Boerner (2009)	Discrepancies in marital quality.	Longitudinal cohort study. CLOC Study: 76 Women 29 Men Age: M = 69	- 9-item Marital Quality Assessment from DAS - CES-D adapted 9-items. - SCL90R adapted 10-items. - Yearning (4-items) and Anger (3-items) from BI, PFL, and TRIG.	- Surviving spouses who rated their marriage more positively than their bereaved partner experienced high levels of anger post-loss (*b* = .84;*p* < .05).	- Physical health. - Depression. - Anxiety. - Caregiving duties.	None.
Carr & Sharp (2014)	Afterlife beliefs.	Longitudinal cohort study. CLOC Study: Sample One: 159 Women 51 Men & Sample Two: 110 Women 45 Men Age: M = 70	- CES-D adapted 9-items. - SCL90R adapted 10-items. - Anger (3-items). - Yearning (4-items). - Intrusive Thoughts (3-items). - Afterlife Beliefs (3-items).	- Uncertain afterlife views predict depression (*b* = 1.09;*p* < .01), anger (*b* = .486;*p* < .10) and intrusive thoughts (*b* = .588;*p* < .05) 6-months post-loss and depression (*b* = .69;*p* < .05) and intrusive thoughts (*b* = .59;*p* < .05), 18-months post-loss.	- Marital quality. - Religious coping. - Physical health. - Economic status. - Gender. - Race. - Age.	- Same sample as Carr et al. (2001), different focus & measures.
Carr, House, Kessler, Nesse, Sonnega & Wortman (2000)	Marital quality (warmth, conflict, & dependence).	Longitudinal cohort study. CLOC Study: Sample One: 150 Women 53 Men Age: M = 73	- CES-D adapted 9-items. - SCL90R adapted 10-items. - Yearning (4-items). - Marital Warmth (7-items), Marital Conflict (2-items), and Marital Dependence (4-items) from DAS.	- High pre-loss marital conflict (*b* = .28;*p* < .01) and lower pre-loss marital warmth (*b* = .17;*p* < .001) results in reduced yearning. - Lower instrumental dependence predicts reduced anxiety (*b* = .27;*p* < .05).	- Spousal health. - Depression. - Anxiety. - Race. - Marital duration. - Social support. - Religiosity. - Self-esteem. - Provision of care pre-death.	None.
Carr, House, Wortman, Nesse & Kessler (2001)	Spousal bereavement context (sudden or expected).	Longitudinal cohort study. CLOC Study: Sample One: 151 Women 59 Men Sample Two: 110 women Age: M = 69 45 men Age: M = 73	- CES-D adapted 9-items. - SCL90R adapted 10-items. - Shock (3-items), Anger (3-items), Yearning (4-items), and Intrusive Thoughts (3-items) from BI, PFL, and TRIG. - Warning Prior to Death (1-item). - Death Context (3-items).	- Prolonged anticipation of spousal death is associated with high anxiety 6-months (*b* = .52;*p* < .05) and 18-months (*b* = .33;*p* < .05) post-loss.	- Physical health. - Depression. - Anxiety.	- Same sample as Carr and Sharp (2014), different focus & measures.
Galatzer-Levy & Bonanno (2012)	Predictors of depression.	Longitudinal cohort study. CLOC study: 269 Women 32 Men Age: M = 72	- CES-D adapted 9-items. - 16-Grief items (yearning, despair, anxiety, shock, and intrusive thoughts) from BI, PFL, and TRIG. - Financial Stress (1-item). - Function Health Index (6-items). - Revised NEOPI adapted 13-items.	- Financial stress is associated with chronic depression (est = .56;SE = .24;*p* < .05) and chronic grief experiences (est = .56;SE = .25;*p* < .05). - Better functional health and scores are associated with resilient (est = -.37;SE = .14;*p* < .01) and depressed-improved responses to spousal loss (est = -1.02;SE = .03; *p* < .001).	Not controlled for.	None.
Ha (2010)	Positive and negative support from adult children.	Longitudinal cohort study. CLOC Study: 148 Participants. Female: 71% Age: M = 73	- CES-D adapted 9-items. - Anger (3-items) and Anxiety (3-items) from BI, PFL, & TRIG. - Positive Support from Children (2-items). - Negative Support from Children (2-items). - Composite Scale (1-item assessing changes in support).	- Positive support from children within 6-months post-loss predicts fewer depressive symptoms 18-months post-loss (*b* = −.16;*p* = .023)	- Suddenness of spousal bereavement. - Co-residence of children. - Attendance at religious services. - Functional limitations.	None.
Ha & Carr (2005)	Parent-child geographic proximity.	Longitudinal cohort study. CLOC Study: 193 bereaved Age: M = 72	- CES-D adapted 9-items. - SCL90R adapted 10-items. - Shock, anger, despair, intrusive thoughts, anxiety, and yearning (19-items) from BI, PFL, and TRIG. - Widowed parent’s social integration 6-months post-loss (2-items). - Parent-Child Geographical Proximity (2-items).	- Widowed parents who live within one hours drive of their children report fewer depressive symptoms (*b* = .59;*p*≤.05) and reduced anxiety (*b* = –.42;*p* ≤ .05) post-loss. - Over-dependence on children is associated with increased depression (*b* = .17;*p* ≤ .05) and anxiety (*b* = .12;*p* ≤ .10)	- Functional limitations. - Support from family and friends	None.
Kim (2009)	Finding meaning in spousal bereavement through religiosity, social support, and caregiving strain.	Longitudinal cohort study. CLOC Study: 91 Women 10 Men Age: M = 72	- Caregiving Strain (1-item). - Finding Meaning (1-item). - Social Support (4-items). - Religiosity (6-items). - Anger (3-items). - Worldview of Acceptance of Death (4-items).	- Finding meaning (*b* = -0.25;*p* < .05) and accepting spousal bereavement (*b* = -0.15;*p* < .05) predicts lower levels of anger. - Increased pre-loss caregiving strain was associated with finding meaning in loss (*b* = .17;*p≤*.05).	Not controlled for.	- Same sample as Kim et al. (2011), same focus & measures.
Kim, Kjervik, Belyea & Choi (2011)	Religiosity, social support, caregiving strain, and finding meaning.	Longitudinal cohort study. CLOC Study: 91 Women 10 Men Age: M = 72	- Caregiving Strain (1-item). - Finding Meaning (1-item). - Social Support (4-items). - Religiosity (6-items). - Personal Strength (3-items).	- Finding meaning in loss predicts personal strength (*b = .38*;*p* < .002). - Social support facilitates finding meaning (*b = .30*;*p* < .001). - Religiosity is linked to higher levels of personal strength (*b = .30*;*p* < .031).	Not controlled for.	- Same sample as Kim (2009), same focus & measures.
Monserud & Markides (2016)	Financial strain, social support, and church attendance.	Longitudinal cohort study. H-EPESE study: 385 bereaved 65% female. Age: M = 72	- CES-D (20-items). - Church Attendance (1-item). - Financial Strain (1-item). - Marital Status (1-item). - Social Support (2-items).	- Greater financial strain predicts higher depression scores (*b = 1.26*;*p < *.01). - More frequent church attendance predicts lower depression scores (*b = .-.25*;*p* < .05). - Greater social support in transition to widowhood predicts greater depression scores (*b = 1.92*;*p* < .05)	Not controlled for.	None.
Pai M & Carr D (2010)	Personality traits.	Longitudinal cohort study. CLOC Study: Sample One: 210 bereaved 87 married controls Sample Two: 159 women 51 men Age: M = 69	- NEOPI (60-items). - CES-D adapted 9-items. - Death Anticipation (1-item).	- Extraversion and conscientiousness predict reduced depression scores (*b = .287*;*p* < .05).	- Demographic characteristics. - Socio-economic resources.	- Same sample as Carr (2003, 2004), different focus & measures.
Prokos & Keene (2005)	Spousal caregiving.	Longitudinal cohort study. CLOC Study: Wave Two: 210 bereaved Female: 71% Age: M = 73 Wave Three. 106 bereaved Female: 91% Age: M = 75	- CES-D adapted 9-items. - Caregiver Stress (1-item). - Level of Care (2-items). - Self-Reported Health (1-item). Emotional Support (2-items).	- Burdensome pre-loss spousal caregiving predicts greater levels of depression *(b* = 0.376;*p≤*.01). - High levels of pre-loss caregiver stress reduces levels of depression (*b* = -0.246;*p≤*.001).	- Pre-existing depressive symptoms.	None.
Sullivan & Infurna (2020)	Engagement and social support.	Longitudinal cohort study. CLOC Study: 265 bereaved Female: 85% Age: M = 70	- CES-D adapted 9-items. - SCL90R adapted 10-items. - Emotional Support (6-items.) - Instrumental Support Given (4-items). - Instrumental Support Received (3-items). - Volunteering (1-item). - Social Integration (3-items). - Religious Involvement (3-items). - Wellbeing (7-items).	- Instrumental support indicated lower levels of depression (*b* = -0.19;*p≤*.05) and greater levels of wellbeing (*b* = 0.14;*p≤*.05) 6- months post-loss.	Not controlled for.	None.

CES-D = Center for Epidemiologic Studies Depression Scale; SCL90R = Symptom Checklist 90; BI = Bereavement Index; PFL = Present Feelings about Loss; TRIG = Texas Revised Inventory of Grief; NEOPI = Neuroticism-Extraversion- Openness-Personality Inventory; DAS = Dyadic Adjustment Scale; EST = Parameter Estimates; SE = Standard Error.

## Results

**Figure 1.**
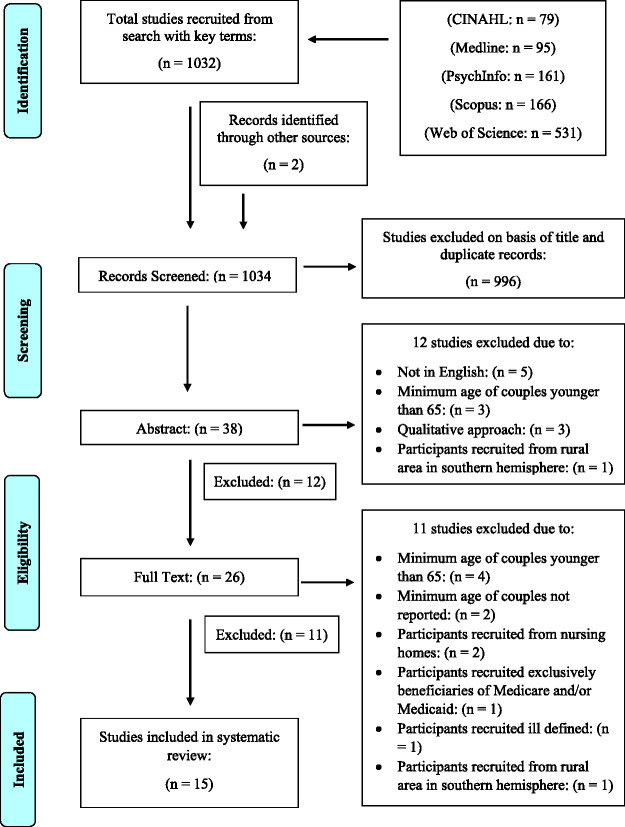
PRISMA Flow Diagram.

The literature search identified 1,034 articles. After removing the duplicates and screening the titles, the abstracts of 38 papers were examined, followed by a careful review of 26 full-test publications. This process identified 15 studies which satisfied criteria for inclusion. This is reflected in a four-phase flow diagram (Figure 1) as recommended by the PRIMSA guidelines for systematic reviews (Moher et al., 2009).

### Quality Appraisal

Study quality was assessed using the Critical Appraisal Skills Programme Cohort Studies Checklist (Critical Appraisal Skills Programme [CASP], 2018; table 1). The CASP (2018) does not advocate an overt scoring system however, the 12-item checklist provides a more holistic all-encompassing quality assessment of longitudinal cohort studies. This is in comparison to appraisal checklists which focus on specific aspects such as methodological quality, for example the Quality Assessment Tool for Observational Cohort and Cross-Sectional Studies (National Heart Lung and Blood Institute, 2014). The inclusion of a more holistic assessment tool was pertinent for this review as several studies gathered data from the same dataset, resulting in overlapping methodological features.

**Table 1.** CASP Checklist for Cohort Studies.

	1	2	3	4	5a	5b	6a	6b	7	8	9	10	11	12
Carr (2003)	✓		✓	✓	✓	✓	✓		✓		N/A	✓	✓	✓
Carr (2004)	✓		✓	✓			✓		✓		N/A	✓	✓	✓
Carr & Boerner (2009)	✓		✓	✓	✓	✓	✓		✓	✓	N/A	✓	✓	✓
Carr & Sharp (2014)	✓		✓	✓	✓	✓	✓	✓	✓	✓	N/A	✓		✓
Carr et al. (2000)	✓		✓	✓	✓	✓	✓		✓	✓	N/A	✓	✓	✓
Carr et al. (2001)	✓		✓	✓	✓	✓	✓	✓	✓	✓	N/A	✓		✓
Galatzer-Levy & Bonanno (2012)	✓		✓	✓			✓	✓	✓		N/A	✓	✓	✓
Ha (2010)	✓		✓		✓	✓	✓	✓	✓	✓	N/A	✓	✓	✓
Ha & Carr (2005)	✓		✓		✓	✓	✓		✓	✓	N/A	✓	✓	✓
Kim (2009)	✓		✓				✓	✓	✓		N/A	✓	✓	✓
Kim et al. (2011)	✓		✓	✓			✓	✓	✓	✓	N/A	✓	✓	✓
Monserud & Markides (2016)	✓	✓	✓				✓	✓	✓	✓	N/A	✓	✓	✓
Pai & Carr (2010)	✓		✓		✓	✓	✓		✓		N/A	✓		✓
Prokos & Keene (2005)	✓		✓	✓			✓	✓	✓		N/A	✓	✓	✓
Sullivan & Infurna (2020)	✓		✓				✓	✓	✓	✓	N/A	✓	✓	✓

Key:

1. Did the study address a clearly focussed issue?

2. Was the cohort recruited in an acceptable way?.

3. Was the exposure accurately measured to minimise bias?

4. Was the outcome accurately measured to minimise bias?

5a. Have the authors identified all important confounding variables? 5b. Have they taken account of the confounding factors in design and/or analysis?

6a. Was the follow-up of subjects complete enough? 6b. Was the follow-up of subjects long enough?

7. What are the results of the study?

8. How precise are the results?

9. Do you believe the results?

10. Can the results be applied to the local population?

11. Do the results of this study fit with other available evidence?

12. Does this study produce implications for practice?

It is worth noting that only 13-items from the CASP checklist were included for evaluation. Item-9 ‘Do you believe the results?’ was excluded on the grounds that science is not a matter of beliefs. Some 12 of the 15 studies (80%) were independently reviewed by a second rater (research assistant GH). The data entry points from each checklist were compared to determine the rate of agreement. Preliminary agreement was 84.6% (132/156). Disagreement was discussed and a consensus was met. Final agreement between raters was 100%.

### Description of Studies

Studies span 20 years of publication from 2000 to 2019, with 14 of 15 studies utilising data from the Changing Lives of Older Couples (CLOC) dataset (Nesse et al., 2003). The remaining study ascertained data from the Hispanic Establish Population for the Epidemiologic Study of the Elderly (H-EPESE) (Markides et al., 2009). The total number of unique participants recruited within this review is 686. This figure includes 385 participants from the H-EPESE dataset utilised within Monserud and Markides’ (2016) study and 301 participants from the CLOC dataset utilised within Galatzer-Levy and Bonanno’s (2012) study. The remaining 13 studies utilised participants which overlapped across studies due to the dominance of the CLOC dataset. Sample sizes varied between 101 and 385, with an average of 214.67 (*SD* = 84.70), and most participants were female (table 2).

### Changing Lives of Older Couples Dataset

The majority of studies utilised the CLOC dataset established by Nesse et al. (2003), which is a multi-wave longitudinal study of spousal bereavement in 1532 married older adults in Detroit, Michigan. Data was collected via face-to-face interviews at baseline and follow-ups which took place between June 1987 and April 1988. The follow-ups included 6-months (wave one), 18-months (wave two), and 48-months (wave three). Of the 319 participants who lost a spouse during the data collection period, 265 participated in at least one interview (Sullivan & Infurna, 2020), 250 participated in interviews at wave one (Carr & Boerner, 2009; Ha & Carr, 2005), 184 interviewed at wave one and two (Carr et al., 2001), and 101 interviewed at all three waves (Kim, 2009; Kim et al., 2011). Data was collected on grief (yearning, despair, anxiety, shock, anger, and intrusive thoughts), financial states, life events, social support, employment, leisure, marriage, family, religion, health, depression, and anxiety. Table 2 outlines the overlapping characteristics between studies.

### Spousal Relationship

Carr (2004) found widows reporting greater pre-loss emotional dependency experienced greater self-esteem post-loss (*b* = .05; *p* < 0.5; N = 297). Sullivan and Infurna (2020) reported greater pre-loss spousal emotional support predicted lower depressive and anxiety symptoms 6-months post-loss (*b* = −.19; *p ≤* .05; N = 265). Separately, Carr (2004) reported widowers with greater pre-loss instrumental dependency experienced increased personal growth post-loss (*b* = −.38; *p* < .05; N = 210). Conversely, Carr et al. (2000) reported greater pre-loss dependency indicated greater post-loss anxiety (*b* = .27; *p* < .01), with greater pre-loss marital conflict (*b* = .16; *p* < .01) and lower pre-loss marital warmth (*b* = .28; *p* < .001) predicting reduced yearning (N = 290). Carr and Boerner (2009) found surviving spouses who rated their marriages more positively than their partner experienced increased anger 6-months post-loss (*b* = .84; *p* < .05; N = 105). Prokos and Keene (2005) found greater pre-loss caregiving stress indicated reduced post-loss depression (*b* = -0.24, *p≤*.001) however, greater pre-loss burdensomeness indicated increased depression 48-months post-loss (*b* = 0.37, *p≤*.01; N = 106).

### Social Support

Ha (2010) reported support from children facilitates psychological adjustment to spousal bereavement 18-months post-loss (*b* = −.16; *p* = .023; N = 148). However, Ha and Carr (2005) reported over-dependence on children is contraindicative of psychological adjustment for both anxiety (*b* = .12; *p* = .10) and depression (*b* = .17; *p* = .05; N = 223). Sullivan and Infurna (2020) reported greater pre-loss instrumental support indicates reduced depression (*b* = .19; *p≤*.05; N = 265) 6-months post-loss. Conversely, Monserud and Markides (2016) reported greater social support in transition to widowhood is associated with increased depression (*b* = 1.92; *p* < .05; N = 385). Finally, Kim (2009) and Kim et al. (2011) reported the importance of social support in facilitating psychological adjustment to spousal bereavement.

### Finding Meaning

Kim (2009) reported finding meaning in spousal bereavement predicted reduced anger post-loss (*b* = .25; *p* < .05; N = 101). Kim et al. (2011) reported that finding meaning is associated with developing personal strength and resilience which contributes to psychological adjustment (*b* = .38; *p* < .002; N = 101). Carr and Sharp (2014) reported uncertain afterlife views predicts increased intrusive thoughts (*b* = .58; *p* < .05), anger (*b* = .48; *p* < .10), and depression post-loss (*b* = 1.09; *p* < .01; N = 155). Finally, Monserud and Markides (2016) found that frequent pre-loss church attendance reduces post-loss depression (*b* = .25; *p* < .05; N = 385).

### Personality Traits

Pai and Carr (2010) found that bereaved older adults who score highly in traits of extraversion and conscientiousness report reduced depression 6-months post-loss (*b* = .28; *p* < .05; N = 210). However, extraversion was not indicative of reduced depression if the death is sudden or unexpected. Additionally, Galatzer-Levy and Bonanno (2012) reported better functional health and scores were associated with resilient (est = -.37; SE = .14; *p* < .01) and depressed improved responses to spousal loss (est = -1.02; SE = .03; *p* < .001; N = 301).

### Death Characteristics

Carr et al. (2001) found prolonged anticipation of spousal death was associated with greater anxiety symptoms both 6 and 18-months post-loss with sudden deaths indicating greater yearning for women only (*b* = .52; *p* < .05; N = 210). Additionally, Carr (2003) found that surviving spouses reported increased intrusive thoughts, anger, and yearning if they perceived their spouse to have experienced a painful death (*b* = .35; *p* < .05; N = 210).

## Discussion

The purpose of this study was to examine the scientific literature on the factors associated with older adults’ psychological adjustment to spousal bereavement. The review identifies certain protective factors that can facilitate a bereaved older adult’s psychological adjustment to spousal bereavement including relationship quality, pre-loss dependency and caregiving stress, social support, finding meaning, personality traits, and death characteristics. These protective factors are mainly associated with lower levels of depressive and anxiety symptoms and reduced ruminative yearning and anger.

Findings which centred around the pre-loss spousal relationship reported that relationships high in conflict, low in warmth, and stressful caregiving experiences contributed to reduced yearning and reduced depression (Carr et al., 2000; Prokos & Keene, 2005). However, increased pre-loss caregiving burdensomeness indicated increased depression (Prokos & Keene, 2005). Insights from the TTM can be drawn upon to conceptualise these findings (Rubin, 1999). Experiences of conflict, lack of care, and stress place high demands on our biopsychosocial resources, the removal of such stressors may contribute to the improvement in biopsychosocial function. Therefore, surviving spouses moving away from these experiences may progress through the biopsychosocial track more proficiently. This would allow for greater internal resources to contend with the relational adjustment to the deceased, resulting in reduced yearning. Alternatively, findings which suggested greater pre-loss burdensome caregiving increased post-loss depression aligned with the ‘wear-and-tear’ theory of care provision which theorizes the accumulative negative impact of caregiving on wellbeing (Townsend et al., 1989). Considering the TTM, surviving spouses who have experienced burdensome caregiving may encounter additional difficulties adjusting to loss. Integral features of the relational track require bereaved individuals to navigate images, memories, and potential negative affect towards the deceased, the loss trajectory, and the upsetting impact on the self system (Rubin et al., 2011). If these relational reflections are complicated due to stressful caregiving experiences, it is understandable for adjustment to be slowed.

The concept that pre-loss dependency indicates an easier adjustment to spousal loss appears counterintuitive. Findings suggest that high levels of pre-loss emotional dependency (widows) and pre-loss instrumental dependency (widowers) each result in increases in self-esteem 6-months post-loss. For widows, Carr (2004) suggested that this boost to self-esteem may result from individuals discovering that they have access to the emotional resources necessary to survive an event that may have previously seemed impossible without the support of their partner. Similarly, for widowers Carr (2004) theorised that individuals may receive a boost in self-esteem by undertaking news responsibilities which align with the traditional expectations of the role of the husband, such as home and financial maintenance. However, Carr et al. (2000) reported contradictory results indicating greater pre-loss spousal dependence increased post-loss anxiety and inhibited psychological adjustment. These differing results may corroborate Neimeyer’s (2001, 2002; 2014) theory of meaning reconstruction. Surviving spouses who successfully reconstruct a sense of order, control, and continuity into their life self-stories which emphasises their ability to cope are likely to experience a boost in self-esteem 6-months post-loss. Whereas surviving spouses who cannot navigate these transitions and fail to maintain a sense of continuity nor readjust to their new relationship with the deceased report more complicated experiences of grief (Neimeyer and Harris, 2016). In this case the complications were reported through increased post-loss anxiety.

Social support is a further construct which presented mixed findings. Ha (2010), Kim (2009), Kim et al. (2011), and Sullivan and Infurna (2020) reported social support as facilitative of psychological adjustment to spousal loss whereas Ha and Carr (2005) and Monserud and Markides (2016) reported social support to be contraindicative of psychological adjustment. These results can be conceptualised according to the DPM in which the oscillation between loss-oriented and restoration-oriented experiences fulfils a crucial role in coping with bereavement (Stroebe & Schut, 2010). Neimeyer and Harris (2016) described this oscillation as the individual ‘dosing’ their exposure to grief. Spending time in restoration-oriented processes with social support can prove to be a useful distraction from grief and therefore support adaptive adjustment. However, it may also be necessary for surviving spouses to ‘dose’ their exposure to social support. If surviving spouses only engage in restoration-oriented processes in the presence of others or feel they cannot engage in loss-oriented experiences in the presence of others, they may experience increases in depression as a result.

Literature examining the concept of finding meaning in spousal loss was unanimous that this process facilitates psychological adjustment (Carr & Sharp, 2014; Kim, 2009; Kim et al., 2011; Monserud & Markides, 2016). Finding meaning is a central tenet of Neimeyer’s (2014) theory of meaning reconstruction following bereavement. Neimeyer and Harris (2016) discuss that bereaved individuals who successfully find meaning in loss can accommodate their loss experience into a life story which provides it with secular, spiritual, or practical significance. This integration of meaning helps individuals to adapt to the bereavement transition and therefore individuals experience less post-loss anger (Kim, 2009) and increased post-loss strength and resilience (Kim et al., 2011).

Extraversion and conscientiousness were associated with psychological adjustment however, neuroticism, prolonged anticipation of death, and the perception that the spouse has died a painful death were indicative of increased depression, anxiety, intrusive thoughts, anger, and yearning post-loss. Individuals scoring high in neuroticism typically respond worse to life stressors and therefore would find it harder to navigate the biopsychosocial track of Rubin’s (1999) TTM. Prolonged anticipation of death could also make it harder for individuals to progress through the relational track of the TTM, as the amount of time invested in preoccupation of loss may influence memories and perceptions of the deceased individual. Finally, bereaved spouses who perceive their loved one experienced a painful death are likely to have increased difficulty finding meaning and making sense of this event. Neimeyer and Thompson (2014) would suggest these individuals will experience greater difficulty accommodating the loss into their life story and subsequently find it more challenging to adjust to the bereavement.

## Limitations

It is worth noting certain limitations. The dominance of the CLOC and H-EPESE datasets leads to results that are based on a relatively small number of participants (n = 686). Additionally, the CLOC dataset commenced recruitment in 1987 with the H-EPESE commencing recruitment in 1993. Therefore, the coping strategies of these cohorts might be different from present-day older adults bereaved by the loss of their partner (Laidlaw, 2015; Satre et al., 2006). The assessment measures utilised throughout the CLOC study are also questionable. For example, Carr (2004), Carr et al. (2000), Ha (2010), Ha and Carr (2005), Kim (2009), Pai and Carr (2010), and Sullivan and Infurna (2020) each utilise assessment measures obtaining Cronbach’s alpha values of poor (<.60), with certain measures reporting internal consistency scores of unacceptable ( < .50). Finally, to be eligible for inclusion within the CLOC dataset participants had to be member of a married couple in which the husband was aged 65 or older. Therefore, there is a possibility that women who lost their husbands during this study were younger than the age of 65 whilst contributing to the dataset. This reduces the validity of the dataset and this review as it cannot categorically be confirmed that all participants contributing to the dataset were classified as an older adult.

Separate to issues of the datasets, grey literature was not included within the search process and therefore it is possible that valuable pieces of research were omitted (Delaney et al., 2005). Additionally, the search, screening, and data extraction processes were completed by one researcher and therefore it is possible that researcher bias could reduce the reliability of the studies synthesised within this review. Finally, the CASP checklist does not provide overall quality scores (CASP, 2018) making it difficult for the quality of these studies to be objectively compared.

## Further Research

Theorists such as Parkes and Prigerson (2013) suggest 6-months post-loss is insufficient to adequately ascertain whether an individual has psychologically adjusted to spousal bereavement, as the grieving processes typically exceeds this. The CASP checklist indicates that longitudinal studies should allow proficient time between baseline and follow-up measurements for desired effects to prominently reveal themselves (CASP, 2018). Therefore, further research examining older adult’s psychological adjustment to spousal bereavement should utilise longer follow-up periods.

The CASP (2018) checklist also highlights the importance of controlling for confounding variables. The impact of this is highlighted in the theme of social support. Monserud and Markides (2016) do not control for confounding variables despite both Ha (2010) and Ha and Carr (2005) controlling for five in total including functional limitations and suddenness of death which impact the form, frequency, and feasibility of social support a bereaved older adult is likely to receive. Consequentially, Ha (2010), and Ha and Carr (2005) report social support to predict positive psychological adjustment to spousal bereavement whereas Monserud and Markides (2016) report greater social support to indicate greater depressive symptoms. Therefore, future research should consider controlling for confounding variables such as religiosity, social support, functional limitations, mental and physical health conditions, demographic characteristics (age and gender), socio-economic resources, bereavement context (provision of care pre-death, suddenness of death), co-residence of children, and pre-loss marital context (duration, quality).

Finally, the literature search revealed additional datasets examining spousal bereavement in older adults from individualistic cultures in the Northern Hemisphere. This included the German Socio-Economic Panel Study (Wagner et al., 2007), the Research and Development (RAND) Health and Retirement Study (RAND, 2011), the Longitudinal Ageing Study Amsterdam (Hoogendijk et al., 2016), and the Living after Loss project (Caserta et al., 2010). However, research utilising these datasets do not report on samples of older adults aged 65 or above. These datasets report on participants aged 50+ or 60+ for example, which reduces their utility in older adult frameworks.

It could be beneficial for academics to draw upon theoretical conceptualisations of ageing, such as Laidlaw’s (2015) model (‘young old’: 65–74, ‘oldest old’: 75+) or the Baltes and Smith (2003) framework (‘third age’: 65–80, ‘fourth age’: 80+). The absence of conceptualisations underpinning the selection of participants across datasets highlights the need to consider this incongruity and strengthen future literature by complying with codified age-related definitions for older adults.

## Conclusions

The aim of this study was to review the empirical literature on the factors associated with older adults’ psychological adjustment to spousal bereavement. Predominantly, findings indicate that this research area requires a greater breadth, depth, and quality of research alongside a movement towards the unification of terminology to further current understanding. However, the findings from this study remain useful for psychological and mental health services.

Foremost, awareness of the protective and risk factors to spousal bereavement will allow bereaved older adults accessing psychological support services to be more proficiently triaged. Therapeutic interventions can be designed and executed whilst holding concepts of post-traumatic growth and psychological adjustment in mind. The concepts of ‘meaning making’ and social support could be integrated as central facets to therapeutic work with service users within this demographic. By identifying older adults who have a large number of ‘risk factors’, early intervention teams could provide more proficient evidence-based support to help balance factors such as perceived burdensomeness, perceived loss of autonomy, and risk of over-dependence on others post-loss, prior to incidents of spousal bereavement.

The information collated could be utilised by practicing clinicians to support older adults experiencing spousal loss. For example, during initial assessments and clinical interviews with surviving spouse’s clinicians should seek greater detail about the pre-loss spousal relationship with reference to emotional and instrumental dependency and whether caregiving duties were undertaken. The context of loss should also be explored to gain insight into whether the death was sudden or expected and whether the surviving spouse perceived their partners death as painful. Clinicians should explore how the surviving spouse is currently engaging in and utilising their social support and pay close attention to the meaning the surviving spouse applies to the events being discussed. Finally, throughout work with older adults who have experienced spousal bereavement clinicians should hold in mind concepts of the DPM (Stroebe & Schut, 2010), the TTM (Rubin, 1999), and most pertinently the theory of meaning reconstruction (Neimeyer, 2001, 2002, 2014).

Future research investigating psychological adjustment to spousal bereavement in older adults is warranted. Further studies should seek to recruit more modern cohorts of older adults, to examine psychological adjustment to spousal bereavement cross-culturally, to specifically examine psychological adjustment to spousal bereavement caused by COVID-19, and to adopt more robust methodological tools and approaches including longer follow-ups and more adequate assessment measures.
